# Role of non-invasive brain stimulation with repetitive transcranial magnetic stimulation in improving fine motor performance of post-stroke patients: a systematic review and meta-analysis

**DOI:** 10.1186/s12883-026-04839-z

**Published:** 2026-04-13

**Authors:** Lisda Amalia

**Affiliations:** Department of Neurology, Medical Faculty Universitas Padjadjaran, Bandung, Indonesia

**Keywords:** Stroke, Fine motor, Rehabilitation, Non-invasive brain stimulation, Transcranial stimulation, Theta-burst stimulation

## Abstract

**Introduction:**

Non-invasive brain stimulation (NIBS) is one of the considered interventions to improve fine motor skills in stroke patients. However, there could be various frequencies and durations performed in each trial involving Non-Invasive Brain Stimulation that might affect the patient’s outcome.

**Objective:**

This research was to investigate the potential enhancement of fine motor performance in stroke patients through the application of non-invasive brain stimulation (NIBS) techniques.

**Methods:**

The literature search was carried out through 3 electronic databases, namely PubMed, Cochrane, and ScienceDirect which include articles published up to 31 December 2024, and published in English. Before selecting the studies, the study duplication was detected by Zotero Reference Manager® and then removed. The related studies examined and selected with rayyan.ai® website using a “blind-on” mode to ensure eligibility.

**Results:**

Twenty-two studies comparing Non-Invasive Brain Stimulation vs. controls with the frequencies of outcomes used were Fugl-Meyer Assessment (68.4%), and Action Research Arm Test (15.7%) were reviewed with thirteen of them included in meta-analysis. All articles were randomized controlled trials. The effect size in repeated transcranial magnetic stimulation (rTMS) stroke comparisons was 2.58 (95% CI, 0.18–4.97; *P* = 0.04; Tau^2^, 19.05; I^2^, 99%; Q, 1367.54; *P* < 0.00001), whereas the Electrical Stimulation for theta burst stimulation (TBS) comparisons was 1.25 (95% CI, 0.01–2.49; *P* = 0.05; Tau^2^, 0.57; I^2^, 54%; Q, 4.34; *P* = 0.11). Conclusion: Non-invasive brain stimulation (NIBS) with rTMS is one of the considered interventions to improve fine motor skills in stroke patients. Non-Invasive Brain Stimulation has a better significance than the conventional therapy methods.

## Introduction

Stroke remains a major global health concern and is one of the leading causes of long-term disability worldwide. Despite advances in acute stroke management, a large proportion of survivors experience persistent neurological deficits that significantly affect functional independence and quality of life. Among these deficits, impairment of upper-limb motor function is particularly common and often limits the ability to perform essential activities of daily living. [1, 48].

Approximately 70–80% of stroke survivors develop upper-limb motor impairment, and many continue to experience long-term deficits in fine motor control. These impairments may affect hand dexterity, coordination, and grip function, which are critical for tasks such as writing, grasping objects, and self-care activities. Consequently, improving upper-limb motor recovery remains a central goal in post-stroke rehabilitation [2].

Conventional rehabilitation approaches, including physiotherapy and occupational therapy, primarily rely on repetitive task-oriented training to facilitate motor recovery. While these interventions can promote functional improvement, recovery outcomes are often incomplete and vary widely among patients. Therefore, additional therapeutic strategies that enhance neuroplasticity and support motor relearning are needed. Non-invasive brain stimulation (NIBS) has emerged as a promising neuromodulatory technique in stroke rehabilitation. Commonly used NIBS modalities include repetitive transcranial magnetic stimulation (rTMS), transcranial direct current stimulation (tDCS), and theta burst stimulation (TBS). These techniques modulate cortical excitability and may facilitate neuroplastic reorganization within motor networks [3–7].

One proposed mechanism underlying NIBS-induced recovery is the restoration of interhemispheric balance between the affected and unaffected hemispheres. After stroke, increased inhibitory activity from the contralesional hemisphere may suppress excitability in the ipsilesional motor cortex, thereby limiting functional recovery. NIBS may help restore this balance by either enhancing excitability in the affected hemisphere or reducing inhibitory signals from the unaffected hemisphere [4].

Although numerous clinical trials have investigated the effects of NIBS on post-stroke motor recovery, the reported outcomes remain heterogeneous due to variations in stimulation protocols, patient characteristics, and rehabilitation approaches [49]. Furthermore, the extent to which different NIBS modalities improve fine motor recovery remains unclear [8, 9, 10, 26].

Therefore, the present systematic review and meta-analysis aimed to evaluate the effectiveness of non-invasive brain stimulation in improving fine motor recovery among stroke patients.

## Methods

### Registration and search strategy

This study was conducted in compliance with the PRISMA guidelines. A thorough search of the PubMed, Cochrane, and ScienceDirect databases from 2019 to 2024 was performed. The main search terms include stroke (“Stroke” or “Post Stroke”) and intervention (“Rehabilitation” or “Intervention”) and (“NIBS”) and fine motor skill (“Fine Motor Skill” or “Fine Motor Skills”) and efficacy (“Efficacy”). Boolean operators were used for combining the search terms.

### Study selection

Following the Population, Intervention, Comparison, and Outcome (PICO) framework, studies were included if they met the following criteria: (1) participants were clinically diagnosed with stroke, with no restrictions on sex, age, disease duration, or severity; (2) the intervention involved non-invasive brain stimulation techniques, including repetitive transcranial magnetic stimulation (rTMS), transcranial direct current stimulation (tDCS), or other forms of transcranial brain stimulation, either alone or combined with rehabilitation approaches such as motor imagery (MI), electrical stimulation (ES), fluoxetine therapy, cervical nerve root magnetic stimulation (CNRMS), or hand-grip training; and (3) the study design was a randomized controlled trial (RCT). Non-invasive brain stimulation (NIBS) is considered a promising neuromodulatory approach for promoting neuroplasticity and facilitating functional recovery after neurological injury. These techniques are non-surgical, relatively easy to administer, and have been increasingly investigated as adjunctive therapies in stroke rehabilitation. In most included studies, sham stimulation was used as the control condition to allow comparison with active stimulation. No restrictions were applied regarding the specific rehabilitation strategies used in the experimental or control groups.

### Data extraction and quality assessment

The following data were extracted independently by the authors:

1. study characteristics, including author, publication year, study design, and country of origin;
2. participant characteristics, including sample size, sex distribution, mean age, stroke duration, stroke type, intervention protocol, supervision, and outcome measures;
3. clinical parameters, including stroke severity assessed using the National Institutes of Health Stroke Scale (NIHSS) and reported statistical outcomes [11];
4. primary outcome, defined as improvement in fine motor function; and.
5. secondary outcomes, including improvements in neural spasticity, activities of daily living (ADL), and quality of life.

When multiple studies were published by the same research group, articles with overlapping study periods or participant populations were carefully examined, and only the most complete dataset was included to avoid duplication.

This systematic review was registered in the PROSPERO International Prospective Register of Systematic Reviews (registration number: CRD4201235106).

### Statistical analysis

Meta-analysis was conducted using a random-effects model to account for potential heterogeneity among the included studies. Effect sizes were calculated as standardized mean differences (SMDs) with corresponding 95% confidence intervals (CIs). Between-study heterogeneity was estimated using the restricted maximum likelihood (REML) method and further quantified using the I2 statistic, which describes the proportion of total variation attributable to heterogeneity rather than chance.

## Results

### Studies Identification

**Fig. 1:**
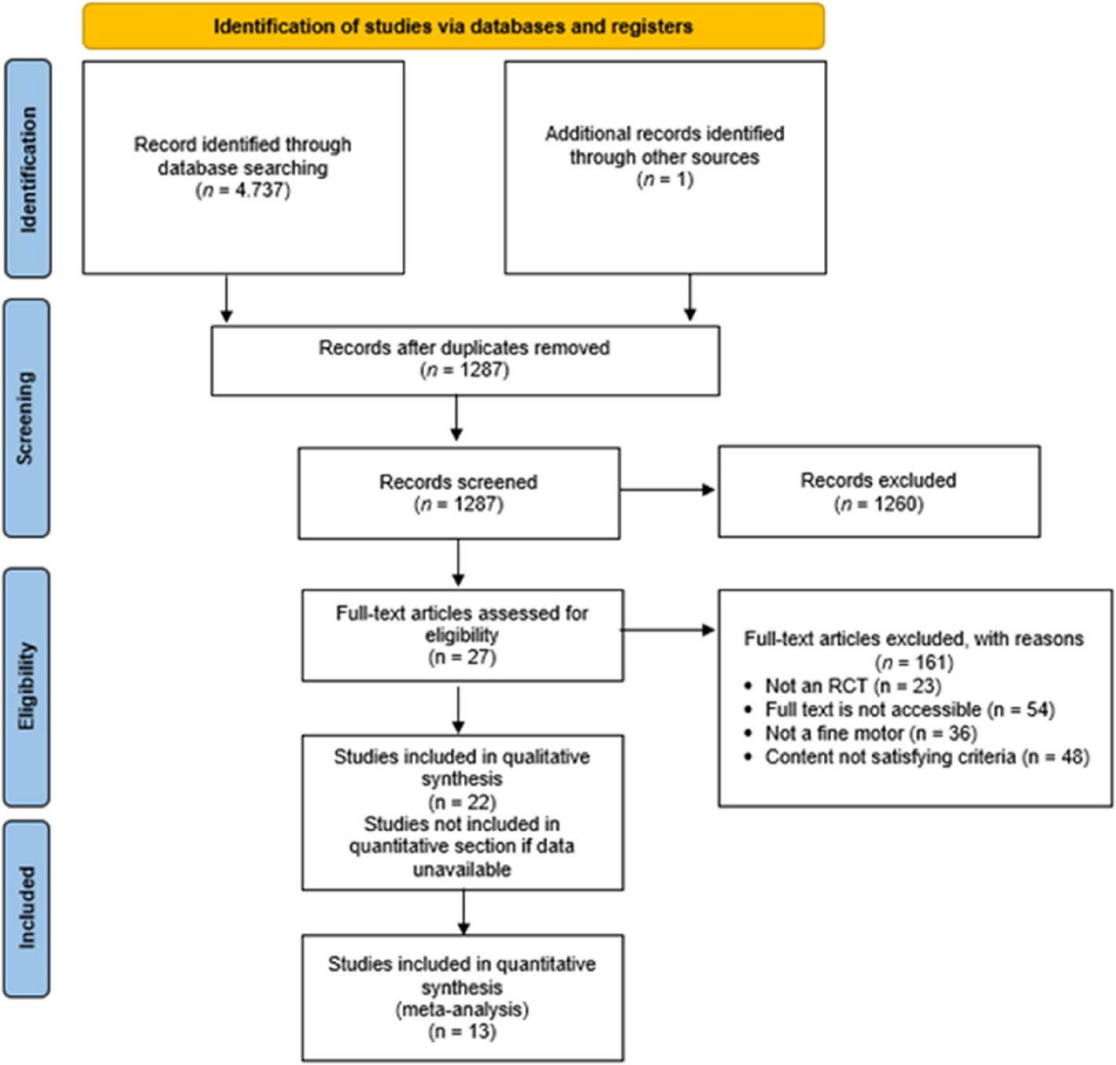
PRISMA flow diagram showing the process of the review

The database search identified 4,738 records, with one additional study identified through other sources. After removing duplicates, 164 studies remained for title and abstract screening. Following the screening process, 27 full-text articles were assessed for eligibility. Ultimately, 22 studies were included in the qualitative synthesis, and 13 studies were included in the meta-analysis. The study selection process is illustrated in the PRISMA flow diagram (Fig. 1).

Twenty-two studies focused on upper extremity rehabilitation [3–9, 12–14]; Quo, 2020; [10, 14–19, 20, 13, 21–23], including arm, hand, and finger paresis and paralysis, whereas one more on the general side with motor impairment. Among them, 8 studies used TMS only [18, 19, 21, 23, 4, 16, 24, 14], 4 studies used transcranial Direct Current Stimulation only [3, 5, 6, 12], 2 studies used Transcranial Brain Stimulation only [7, 17], and the remaining 8 used combined interventions [7, 13, 8]; Quo, 2020; [10, 20, 22, 25]. For combined treatment, we included interventions that are combined with Non-Invasive Brain Stimulation, such as Motor Imagery (MI), Electrical stimulation (ES), Fluoxetine, Cervical Nerve Root Magnetic Stimulation (CNRMS), and hand grip training. All interventions’ duration varied from one to two weeks with 5 to 10 consecutive sessions. Table 1 summarizes these principles.

**Table 1:** Population characteristics

Journal Data		Population Characteristics								Intervention Detail	
Study	Location	Sample size	Age (mean ± SD)	Gender Male, n (%)	Type of Stroke (%)		Staging of Stroke	Severity	Type of Fine Motor Dysfunction	NIBS Parameters	Outcome
					Ischaemic	Hemorrhagic					
Ibrahim et al. [4]	Egypt	40	59.45 ± 5.46	24 (60)	100	0	Subacute and chronic	severe	left upper extremity paresis	(1) rTMS M1, (2) s-rTMS M1, 10 consecutive sessions × 5 Hz x 87 dB × 80% MSO	FMA-UE
Boasquevisque et al. [3]	Brazil	30	61.85 ± 16.45	12 (40)	100	0	subacute	moderate	upper limb paresis	(1) IL c-tDCS M1, (2) s-tDCS. 6 sessions x 1 mA × 20 min × 35 cm2 sponge	NIHSS, FMA, mRS, BI, MAS
Bolognini et al. [5]	Italy	32	68.5 ± 10.75	21 (65.63)	78.13	21.87	NA	severe	motor impairment	(1) IL a-tDCS M1, (2) CL c-tDCS M1. 10 session × 2 mA × 15 min × 35 cm2 sponge	MI-UL, NIHSS, BI
Bornheim et al. (2019)	Belgium	50	62.98 ± 12.4	33 (66)	100	0	Acute, subacute, and chronic	mild to moderate	hand paresis	(1) CL a-tDCS, (2) s-tDCS. 20 sessions x 2 mA × 20 min × 25 cm2 surface electrodes	WMFT, BI, SIS
Chen et al. [7]	Taiwan	22	52.75 ± 9.7	14 (63.64)	22.73	77.27	chronic	moderate	hemiplegia and hemiparesis	(1) iTBS M1, (2) sham. 5 consecutive sessions × 50 Hz × 600 p/s × 80% AMT	ARAT, MAS, FMA-UE, MAL, BBT
Edwards et al. [12]	USA	82	67.8 ± NA	50 (60.98)	NA	NA	chronic	mild to severe	arm hemiparetic	(1) IL a-tDCS M1, (2) s-tDCS. 36 session × 2 mA × 20 min × 35 cm2 sponge	FMA-UE
Garrido et al. (2022)	Chile	70	65 ± 13	37 (52.86)	97.14	2.86	acute	moderate	upper limb paralysis	(1) BL-tDCS (IL anode and CL cathode M1) + CIMT, (2) sham. 14 session × 2 mA × 20 min × 25 cm2 sponge	FMA-UE
Kang et al. [13]	South Korea	20	64.94 ± 9.38	10 (58.82)	88.24	11.76	subacute	mild	upper extremity paresis	(1) rTMS + MI + ES, (2) s-rTMS + MI. 10 sessions × 1 Hz × 1200 p/s × 90% of RMT	MMSE, FMA, MBI, Purdue Pegboard
Ke et al. [21]	China	48	56.5 ± 8.6	20 (41.67)	100	0	acute	moderate	upper limb paralysis	(1) HF a-rTMS M1, (2) s-rTMS M1, 10 consecutive sessions × 20 Hz × 1200 p/s × 110% of RMT	FMA-UE
Kim et al. [19]	South Korea	73	62.06 ± 12.16	45 (61.64)	100	0	NA	mild	upper limb dysfunction	(1) rTMS M1, (2) s-rTMS M1, 10 consecutive sessions × 1 Hz × 100% of RMT (pulse not clearly described)	FMA-UE
Kuo et al. [26]	Taiwan	18	61 ± 12.14	9 (50)	100	0	subacute	mild to moderate	hand paresis	(1) BL-tDCS (IL anode and CL cathode M1) + TMS, (2) BL-tDCS (IL anode and CL cathode M1) + MEG, (3) s-tDCS + TMS, (4) s-tDCS + MEG. 2 sessions × 2 mA × 20 min × 35 cm2 sponge	FMA-UE, NIHSS, mRS, MRC, ARAT
Liu et al. [23]	China	58	58.12 ± 6.75	26 (44.83)	60.34	39.66	chronic	mild to severe	motor impairment	(1) 10 Hz rTMS × 700 pulses × 90% of RMT × 10 sessions (2) s-rTMS	FIM
Luk et al. [18]	China	24	66.2 ± 4.45	14 (58.33)	95.83	1.47	chronic	mild to moderate	Paretic hand and limb	(1) rTMS M1, (2) s-rTMS M1, 10 consecutive sessions × 1 Hz × 1200 p/s × 90% of rMT	NHPT, ARAT, FMA-UE, BBT
Meng et al. [25]	China	28	53.84 ± 10.56	18 (64.28)	57.14	42.86	Subacute and chronic	moderate	hand paretic	(1) CL rTMS M1 + IL iTBS M1, (2) Cl rTMS M1 + IL s-iTBS, (3) CL s-rTMS + IL s-iTBS. 1 Hz rTMS × 1200 p/s × 100% of RMT. 10 sessions × 50 Hz iTBS × 1200 p/s × 80% of RMT	FMA-UE
Pinto et al. [20]	USA	27	54.78 ± 12.25	16 (59.26)	100	0	Chronic	moderate	hemiplegia/hemiparesis	(1) Fluoxetine + rTMS, (2) Fluoxetine, (3) s-rTMS. 10 sessions × 1 Hz × 1200 p/s × 100% of RMT	NIHSS, JTHFT, FMA-UE, MMSE, HDRS
Qin et al. [9]	China	49	58.59 ± 9.89	31 (63.27)	100	0	Chronic	moderate	upper limb dysfunction	(1) LF-rTMS M1 + rPMS, (2) LF-rTMS (3) CG. 3 sessions x 1 Hz × 90% RMT × 1,200 p/s	FMA-UE, MAS, MBI (other outcome)
Rosso et al. [16]	France	27	61.56 ± 12.56	21 (77.78)	100	0	Chronic	mild to moderate	upper limb dysfunction	(1) rTMS M1, (2) s-rTMS. 5 consecutive sessions × 0.2 Hz × 90% of RMT (conditioning stimulus) and 140% of RMT (test stimulus)	FMA-UE
Vink et al. [17]	Netherland	59	60.2 ± 12	40 (68)	85	15	Subacute	moderate	arm paretic	(1) cTBS M1, (2) s-cTBS. 10 consecutive sessions × 50 Hz × 70% of RMT (pulse not clearly described)	JTHFT, NHPT, ARAT, FMA-UE, SIS
Wang et al. [14]	China	45	59.87 ± 12.26	30 (66.67)	66.67	33.33	Subacute	severe	motor impairment	(1) HF rTMS M1, (2) LF rTMS, (3) s-rTMS M1, 14 consecutive sessions × 10 Hz × 1000 p/s × 100% of RMT	FMA-UE
Wang et al. [24]	China	29	55.41 ± 7.48	18 (62.07)	65.5	34.5	Subacute	mild to moderate	upper limb dysfunction	(1)LF rTMS M1, (2) s-rTMS M1. 10 sessions × 1 Hz × 90% of RMT (pulse not clearly described)	FMA-UE, WMFT, MBI (other outcome)
Wu et al. [8]	China	60	55.45 ± 10.96	48 (80)	45	55	Subacute and chronic	moderate	upper limb dysfunction	(1) rTMS M1 + CNRMS (2) rTMS (3) CNRMS (4) s-rTMS M1. 10 consecutive sessions × 10 Hz × 1.000 p/s × 80% of RMT	FMA-UE
Yang et al. [10]	China	39	62.92 ± 8.72	28 (71.79)	79.49	20.51	Subacute and chronic	moderate	upper limb and hand dysfunction	(1) HF rTMS + hand grip training M1, (2) HF rTMS (3) hand grip training.10 consecutive sessions × 5 Hz × 750 p/s × 100% of RMT	FMA-UE

*a-tDCS* anodal transcranial direct current stimulation, *BL-tDCS* bilateral transcranial direct current stimulation, *CIMT* constraint induced movement therapy, *CL* contralesional, *c-tDCS* cathodal transcranial direct current stimulatio, *IL* ipsilesional, *M1* primary motor cortex, *N.A* not available, *NIBS* non-invasive brain stimulation, *p/s* pulses/session, *RMT* resting motor threshold, *AMT* active motor threshold, *rPNS* repeated peripheral nerve stimulation, *rTMS* repeated transcranial magnetic stimulation, *s-rTMS* sham repeated transcranial magnetic stimulation, *HF* high frecuency, *LF* low frequency, *s-tDCS* sham transcranial direct current stimulation, *tDCS* transcranial direct current stimulation, *FMA-UE* fugl-meyer assessment for upper extremity, *JTHFT* jjebsen-taylor hand function test, *ARAT* action research arm test, *NHPT* nine hole peg test, *NIHSS* national institutes of health stroke scale, *BBT* box block test, *MAS* modified ashworth Scale, *BI* barthel index, *MBI* modified barthel index, *WMFT* wolf motor function test, *mRS* modified rankin scale, *MRC* medical research council scale, *SIS* Stroke Impact Scale, *MMSE* mini-mental state examination, *HDRS* hamilton depression rating scale, *CNRMS* cervical nerve root magnetic stimulation, *CG* control group, *MSO* maximal stimulator output, Data are given as n and mean ± SD

### Study characteristics

The included studies primarily investigated interventions targeting upper-limb motor recovery in stroke patients. Among the 22 studies: 8 studies evaluated rTMS, 4 studies evaluated tDCS, 2 studies used other neuromodulation techniques, 8 studies investigated combined interventions. The intervention duration ranged from 1 to 2 weeks, typically involving 5–10 treatment sessions. Several studies combined NIBS with conventional rehabilitation approaches, including: physiotherapy, occupational therapy, motor imagery training, electrical stimulation. Further characteristics are detailed in Table 1.

### Risk of bias

**Fig. 2:**
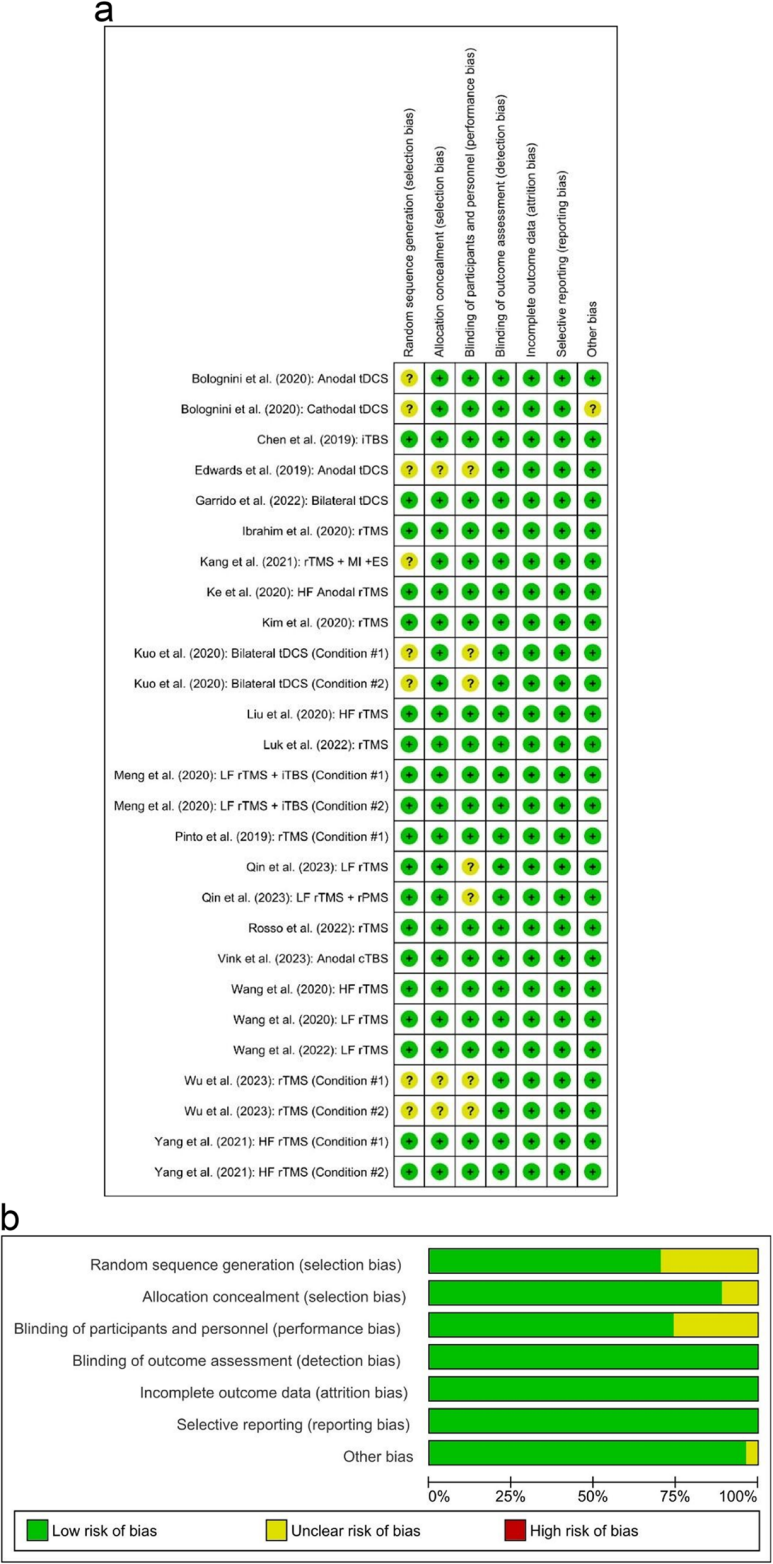
Quality of the included randomized controlled trials. **a** Risk-of-bias summary: review authors’ judgments about each risk-of-bias item for each included study. **b** Risk-of-bias graph: review authors’ judgments about each risk-of-bias item presented as percentages across all included studies. Forrest plot of non-invasive brain stimulation paradigms

With the Revised Cochrane risk-of-bias tool for randomized trials (RoB 2), the overall risk of bias was low in sixteen studies, whereas three studies had some concerns, and three had a high risk of bias. Overall, the methodological quality of the included studies was considered acceptable. Figure 2 summarizes the Revised Cochrane risk-of-bias tool for randomized trials (RoB 2) results of the studies.

## Fine motor recovery measurements

**Fig. 3:**
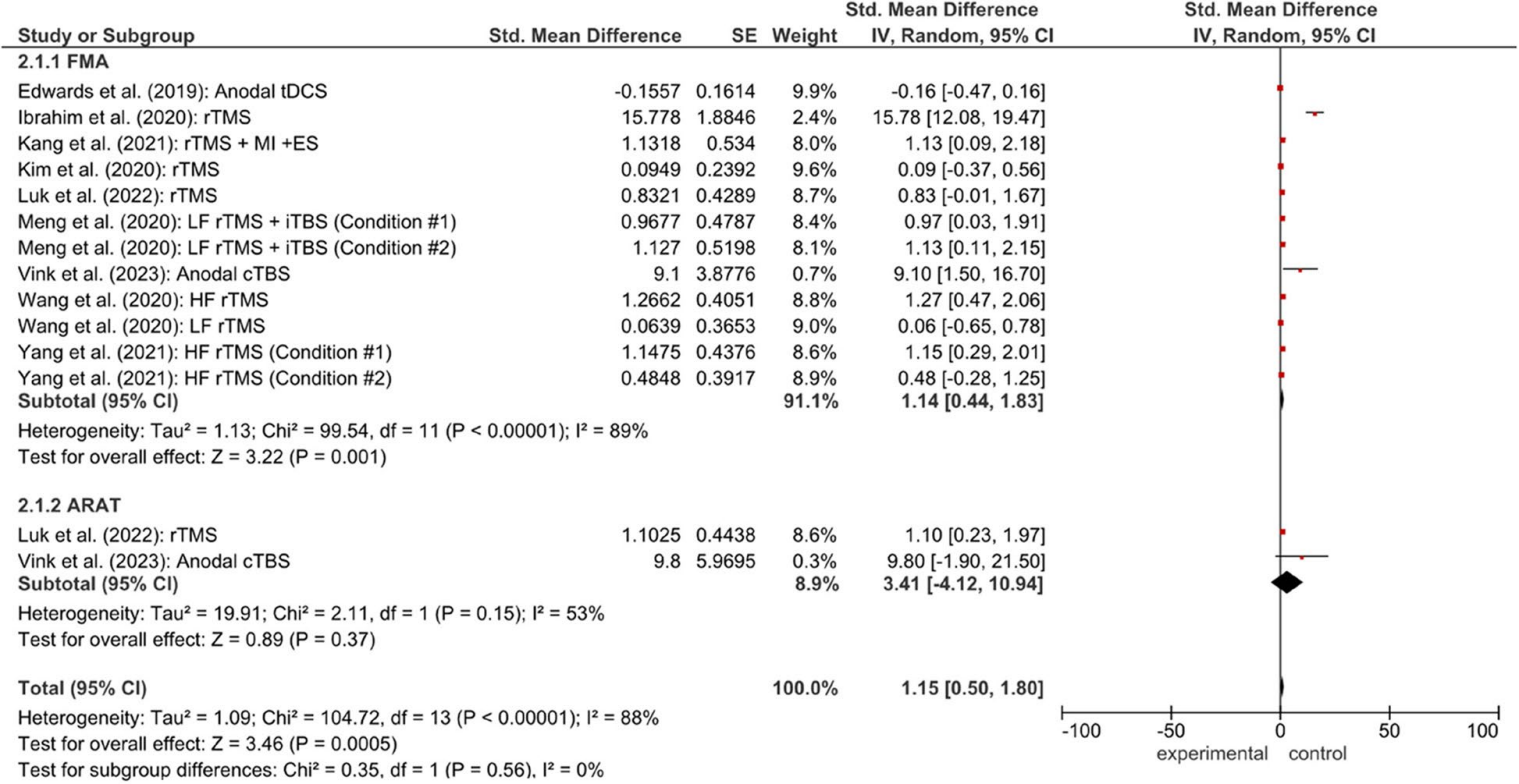
Forest plot for the effectiveness of NIBS of fine motor in subacute and chronic stroke patients

**Fig. 4:**
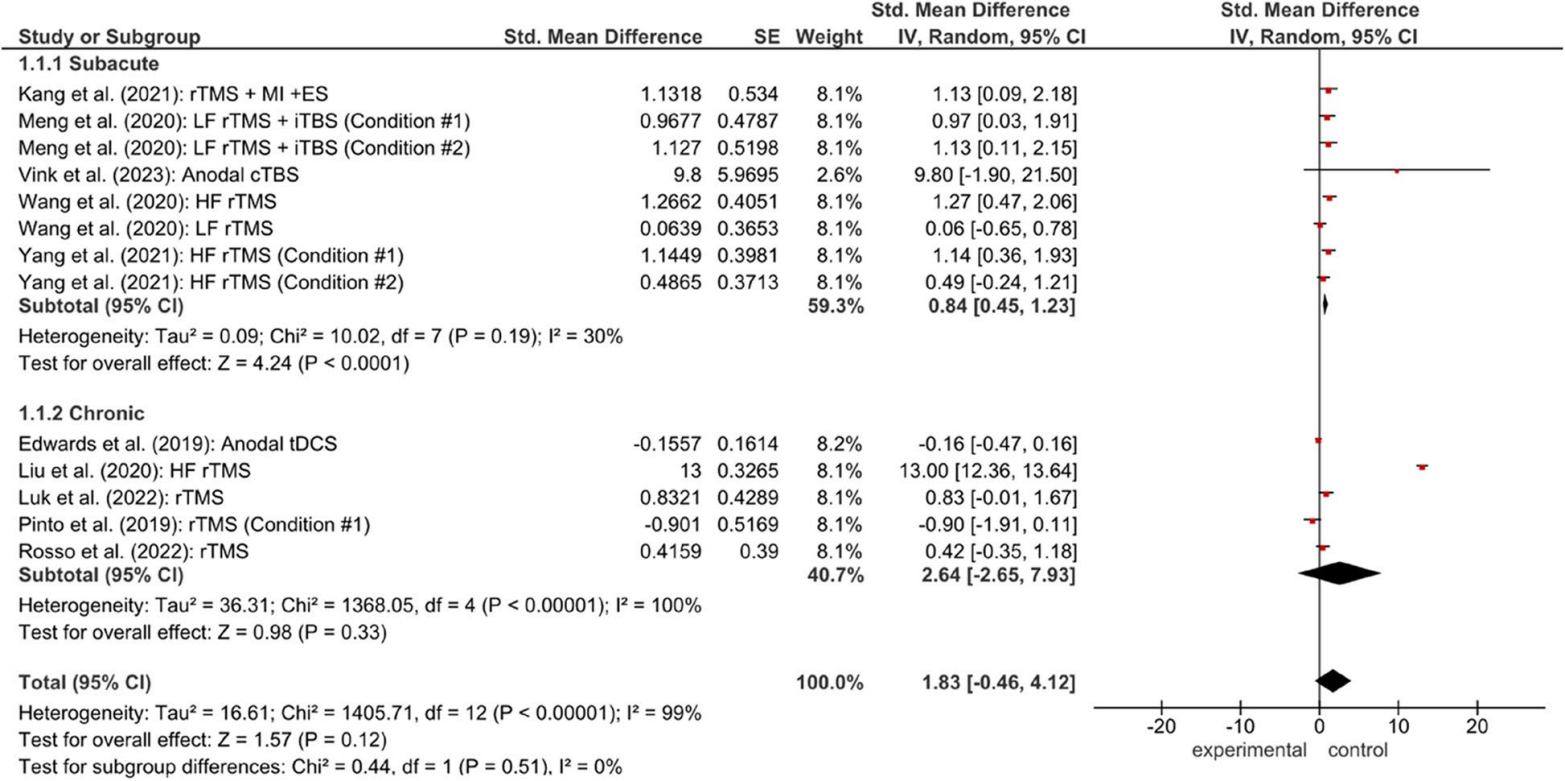
Forest plot for the effectiveness of assessment of fine motor in FMA and ARAT. Information for condition #1 and condition #2 in Meng et al., [25] and Yang et al., [10] is provided in table characteristics. Heterogeneity calculated by Restricted Maximum Likehood-method. Abbreviation: CI, confidence interval,df, degrees of freedom; SE, standard error; iTBS, intermittent theta burst stimulation; IV, inverse variance; rTMS, repeated transcranial magnetic stimulation; tDCS, transcranial direct current stimulation; HF, high frequency; LF, low frequency; FMA, fugl-meyer assessment; ARAT, action research arm test

In assessing fine motor skills, several instruments are used, namely FMA-UE, Fugl-Meyer Assessment for Upper Extremity; JTHFT, Jebsen-Taylor hand function test; ARAT, Action Research Arm Test; NHPT, Nine-Hole Peg Test; NIHSS, National Institutes of Health Stroke Scale; BBT, Box and Block Test; BI, Barthel Index; MBI, Modified Barthel Index; WMFT, Wolf Motor Function Test; mRS, modified Rankin Scale; MMSE, Mini-Mental State Examination; HDRS, Hamilton Depression Rating Scale. The most used assessment tool is FMA (13 studies), followed by ARAT (4 studies). Figures 3 and 4 have a detailed summary of each study and a simplified checklist**.**

### Effectiveness analysis of non-invasive brain stimulation in repeated transcranial magnetic stimulation and transcranial brain stimulation

The pooled results demonstrated that non-invasive brain stimulation significantly improved fine motor recovery in stroke patients. Subgroup analysis showed that rTMS significantly improved motor recovery in both subacute (effect size = 2.58, 95% CI = 0.18–4.97) and chronic stroke patients (effect size = 3.34). However, heterogeneity across studies was substantial.

In contrast, tDCS showed significant improvement when combined with conventional rehabilitation therapies (Table 2). Overall, Non-Invasive Brain Stimulation intervention showed high effectiveness in improving fine motor function of stroke patients (Table 3).

**Table 2:** Improvement Detail

Journal Data		Pre Intervention			Post Intervention		
Study	Stroke onset, days (mean ± SD)	Severity	Parameters	Baseline	Severity	Parameters	Outcome
Ibrahim et al. [4]	NA	severe	NIHSS	MRC Intervention group: 1.50 ± 0.44 Comparator group: 1.59 ± 0.45 FMA-UE Intervention group: 18.15 ± 4.43 Comparator group: 17.30 ± 5.68	NA	NA	MRC Intervention group: 2.70 + −0.44 Comparator group: 1.79 + −0.50 FMA-UE Intervention group: 33.85 + −5.28 Comparator group: 18.30 + −6.59
Boasquevisque et al. [3]	7—90 (they only give median for the specific)	moderate	NIHSS	Median(IQR) Primary Outcome FMA Intervention group: 46 (8; 56.8) Comparator group: 22.5 (8.8; 43.5) Secondary Outcome mRS Intervention group: 3 (2; 4) Comparator group: 4 (3; 4) BI Intervention group: 80 (47.5; 95) Comparator group: 65 (47.5; 77.5) MASshoulder Intervention group: 0 (0; 1) Comparator group: 0 (0; 1) MASelbow Intervention group: 0 (0; 1.25) Comparator group: 1 (0; 2) MASwrist Intervention group: 0.5 (0; 2.25) Comparator group: 1 (0.8; 2) MASfingers Intervention group: 0.5 (0; 1.25) Comparator group: 1 (0; 1) MALquantitative Intervention group: 1.05 (0; 1.97) Comparator group: 0.1 (0; 0.4) MALqualitative Intervention group: 0.89 (0; 1.67) Comparator group: 0 (0; 0.2)	Intervention group: mild Comparator group: moderate	NIHSS	Median(IQR) Primary Outcome FMA Intervention group: 51(16,8;61,5) Comparator group: 38.5 (20.5; 55.8) Secondary Outcome mRS Intervention group: 3 (2; 4) Comparator group: 3 (3; 3) BI Intervention group: 85 (57.5; 100) Comparator group: 77.5 (67.5; 90) MASshoulder Intervention group: 0 (0; 0) Comparator group: 0 (0; 0) MASelbow Intervention group: 0.5 (0; 1) Comparator group: 1 (0; 2) MASwrist Intervention group: 0 (0; 1.25) Comparator group: 1 (0; 2) MASfingers Intervention group: 0 (0; 1) Comparator group: 0 (0; 1) MALquantitative Intervention group: 2.41 (0; 3.5) Comparator group: 0.6 (0; 1.9) MALqualitative Intervention group: 2.16 (0; 3.58) Comparator group: 0.7 (0; 1.3)
Bolognini et al. [5]	NA	moderate	NIHSS	Primary Outcome MI-UL Intervention group: 26.4 ± 28.4 Comparator group: 31.8 ± 31.6 Secondary Outcome HGS Intervention group: 3.3 ± 6.5 Comparator group: 4.9 ± 8.7 BI Intervention group: 19.4 ± 27.9 Comparator group: 24.7 ± 32.3	NA	NIHSS	NA (graph)
Bornheim et al. (2019)	1—365	NA	NA	WMFT handgrip strength Intervention group: 18.2 ± 8.24 Comparator group: 17.92 ± 9.7 Tardieu Intervention group: 0 Comparator group: 0 Barthel Index Intervention group: 61.6 ± 21.3 Comparator group: 64.4 ± 19.54 SIS Intervention group: 176.12 ± 95.79 Comparator group: 190.84 ± 93.34 FM-UE NA (graph) FM-LE NA (graph)	NA	NA	NA (graph)
Chen et al. [7]	≥ 180	moderate	NIHSS	MAS-UE Intervention group: 3.90 ± 2.10 Comparator group: 4.05 ± 1.56 FMA-UE Intervention group: 33.33 ± 19.80 Comparator group: 30.03 ± 22.11 ARAT Intervention group: 18.62 ± 19.38 Comparator group: 16.72 ± 22.8 ARAT Grasp Intervention group: 4.00 ± 4.48 Comparator group: 3.52 ± 5.28 ARAT Grip Intervention group: 4.80 ± 7.08 Comparator group: 5.58 ± 7.86 ARAT Pinch Intervention group: 2.46 ± 3.39 Comparator group: 1.86 ± 3.51 BBT Intervention group: 10.20 ± 16.80 Comparator group: 9.00 ± 21.00 MAL (AOU) Intervention group: 10.44 ± 16.38 Comparator group: 18.9 ± 22.96 MAL (QOM) Intervention group: 10.50 ± 13.44 Comparator group: 15.26 ± 22.40	NA	NIHSS	MAS-UE Intervention group: 3.30 ± 1.98 Comparator group: 4.29 ± 1.44 FMA-UE Intervention group: 34.65 ± 19.80 Comparator group: 27.06 ± 20.79 ARAT Intervention group: 23.18 ± 20.71 Comparator group: 13.3 ± 19.95 ARAT Grasp Intervention group: 4.60 ± 4.96 Comparator group: 2.68 ± 4.72 ARAT Grip Intervention group: 6.42 ± 7.20 Comparator group: 5.04 ± 7.38 ARAT Pinch Intervention group: 3.51 ± 3.21 Comparator group: 1.26 ± 2.82 BBT Intervention group: 11.40 ± 18.60 Comparator group: 9.60 ± 22.20 MAL (AOU) Intervention group: 12.18 ± 14.70 Comparator group: 20.72 ± 22.54 MAL (QOM) Intervention group: 10.36 ± 13.86 Comparator group: 21.28 ± 22.12
Edwards et al. [12]	1317	NA	NA	Primary Outcome FM Intervention group: 25.7 ± 16.3 Comparator group: 25.3 ± 16.3 Secondary Outcome WMFT Intervention group: 56.0 (47.2) Comparator group: 60.0 (48.3)	NA	NA	Primary Outcome FM Intervention group: 33.4 +—19.2 Comparator group: 32.0 +—18.8 Secondary Outcome WMFT Intervention group: 68.5 (23.2) Comparator group: 67.1 (54.0)
Garrido et al. [22]	6 +—4	moderate	NIHSS	Primary Outcome FMA-MOTOR FUNCTION Intervention group: 45.9 ± 12.1 Comparator group: 44.0 ± 12.9 Secondary Outcome WMFT-FAS Intervention group: 44 ± 19 Comparator group: 42 ± 19 FIM Intervention group: 70 ± 22 Comparator group: 69 ± 21	NA	NA	Primary Outcome FMA-MOTOR FUNCTION Intervention group: 58.7 8.0 Comparator group: 53.4 9.6 Secondary Outcome WMFT-FAS Intervention group: 63.5 15.6 Comparator group: 56.9 15.5 FIM Intervention group: 113.7 11.4 Comparator group: 103.4 20.9
Kang et al. [13]	26.01 ± 16.92	NA	NA	Primary Outcome FMA Intervention group: 28.13 ± 22.69 Comparator group: 29.78 ± 20.20 Secondary Outcome SAFE Intervention group: 4.50 ± 2.07 Comparator group: 4.44 ± 1.59 MBI Intervention group: 55.38 ± 14.78 Comparator group: 49.22 ± 20.28 Perdue Pegboard Intervention group: 1.13 ± 1.89 Comparator group: 1.00 ± 2.12 Finger Tapping Test Intervention group: 8.25 ± 10.04 Comparator group: 7.11 ± 13.07	NA	NA	Primary Outcome FMA Intervention group: 39.88 ± 23.31 Comparator group: 33.44 ± 21.68 Secondary Outcome SAFE Intervention group: 5.63 ± 1.92 Comparator group: 4.89 ± 1.90 MBI Intervention group: 70.25 ± 16.25 Comparator group: 61.22 ± 18.13† Perdue Pegboard Intervention group: 2.75 ± 3.15 Comparator group: 1.22 ± 2.54 Finger Tapping Test Intervention group: 14 ± 12.57 Comparator group: 12.11 ± 15.66
Ke et al. [21]	12.05 ± 2.61	NA	NA	Primary Outcome FMA UE Long ITI rTMS group: 43.4 ± 21.3 Short ITI rTMS group: 34.5 ± 12.4 Sham group: 41.5 ± 20.5 Secondary Outcome BI Long ITI rTMS group: 54.1 ± 17.6 Short ITI rTMS group: 52.4 ± 21.1 Sham group: 45.6 ± 22.3	NA	NA	NA (graph)
Kim et al. [19]	NA	mild	NIHSS	Primary Outcome BBT (median, IQR) Intervention group: 17 ± 28 Comparator group: 13 ± 27 Secondary Outcome FMA Intervention group: 41.2 ± 17.7 Comparator group: 40.2 ± 18.0 FTT (median, IQR) Intervention group: 14 ± 23 Comparator group: 13 ± 22 MAS Intervention group: 0 ± 0 Comparator group: 0 ± 1	Moderate	NIHSS	Only shows changes data in outcome
Kuo et al. [26]	0.69 ± 0.18 23 (median)	NA	NA	NA (graph)	NA	NA	NA (graph)
Liu et al. [23]	261.3 ± 55.2	NA	NA	Primary Outcome FIM Intervention Group: 66.21 Comparator Group: 63.83 Secondary Outcome MMSE Intervention Group: 15.55 Comparator Group: 15.31 DST Intervention Group: 4.83 Comparator Group: 4.55 DS Intervention Group: Comparator Group:	NA	NA	Primary Outcome FIM Intervention Group: 83.90 Comparator Group: 69.55 Secondary Outcome MMSE Intervention Group: 18.62 Comparator Group: 16.51 DST Intervention Group: 8.59 Comparator Group: 5.31 DS Intervention Group: Comparator Group:
Luk et al. [18]	107.7 ± 196.5	NA	NA	Primary Outcome MEP Intervention Group: 258.8 Comparator Group: 600.1 IHA Intervention Group: −0.39 Comparator Group: −0.06 FMA Intervention Group: 46.7 Comparator Group: 48.8 Secondary Outcome ARAT Intervention Group: 36.5 Comparator Group: 41.1 BBT Intervention Group: 17.2 Comparator Group: 33.8 RT Intervention Group: 985.8 Comparator Group: 654.1	NA	NA	Primary Outcome MEP Intervention Group: 369.1 Comparator Group: 586 IHA Intervention Group: 0.04 Comparator Group: −0.21 FMA Intervention Group: 57.0 Comparator Group: 53.3 Secondary Outcome ARAT Intervention Group: 51.1 Comparator Group: 43.2 BBT Intervention Group: 34.8 Comparator Group: 41.8 RT Intervention Group: 837.3 Comparator Group: 637.9
Meng et al. [25]	34.8 ± 223.5	moderate	NIHSS	Primary Outcome UE-FMA (changes) Secondary Outcome BI (changes) MEP amplitude Real 1 Hz rTMS-Real iTBS: 1.14 ± 0.72 Real 1 Hz rTMS-Sham iTBS: 0.97 ± 0.33 Sham 1 Hz rTMS-Sham iTBS: 0.82 ± 0.24 MEP latency Real 1 Hz rTMS-Real iTBS: 24.5 ± 1.7 Real 1 Hz rTMS-Sham iTBS: 23.9 ± 1.7 Sham 1 Hz rTMS-Sham iTBS: 25.4 ± 1.5			Primary Outcome UE-FMA (changes) Secondary Outcome BI (changes) MEP amplitude Real 1 Hz rTMS-Real iTBS: 2.57 ± 0.63 Real 1 Hz rTMS-Sham iTBS: 1.87 ± 0.64 Sham 1 Hz rTMS-Sham iTBS: 1.22 ± 0.25 MEP latency Real 1 Hz rTMS-Real iTBS: 21.4 ± 2.1 Real 1 Hz rTMS-Sham iTBS: 22.3 ± 1.6 Sham 1 Hz rTMS-Sham iTBS: 25.0 ± 1.5:
Pinto et al. [20]	291.41 ± 239.84	moderate	NIHSS	Primary Outcome JTHF Combined: 526.91 (279.92) Fluoxetine: 594.06 (195.28) Placebo: 439.39 (306.43) FMA Combined: 27 (14.89) Fluoxetine: 25.67 (11.67) Placebo: 33.75 (14.68) Secondary Outcome MAS Combined: NA Fluoxetine: NA Placebo: NA BDI median (min–max) Combined: 7 (0–30) Fluoxetine: 4 (0–12) Placebo: 11 (3–14) MMSE Combined: 29.11 (1.36) Fluoxetine: 29.38 (1.41) Placebo: 29 (1.2) HDRS Combined: 5.77 (3.7) Fluoxetine: 4.3 (3.02) Placebo: 6.75 (3.81)	NA	NIHSS	NA (Changes)
Qin et al. [9]	96 ± 54.3	NA	NA	MAS Intervention Group: 1.76 ± 0.45 Comparator Group: 1.78 ± 0.51 FMA UE Intervention Group: 25.33 ± 9.91 Comparator Group: 23.64 ± 7.61 MBI Intervention Group: 35.13 ± 16.25 Comparator Group: 32.14 ± 13.41	NA	NA	MAS Intervention Group: 1.53 ± 0.48 Comparator Group: 1.57 ± 0.64 FMA UE Intervention Group: 35.26 ± 7.43 Comparator Group: 28.57 ± 7.00 MBI Intervention Group: 50.80 ± 14.27 Comparator Group: 341.21 ± 12.49
Rosso et al. [16]	8640 ± 12,540 (days)	NA	NA	Primary Outcome JTHFT JTHFT Intervention Group: 5.92 ± 6.95 Comparator Group: 9.03 ± 11.7 Secondary Outcome GS GS Intervention Group: 0.37 ± 0.27 Comparator Group: 0.37 ± 0.26	NA	NA	Primary Outcome JTHFT JTHFT Intervention Group: 5.31 ± 6.66 Comparator Group: 10.14 ± 12.38 Secondary Outcome GS GS Intervention Group: 0.53 ± 0.27 Comparator Group: 0.41 ± 0.29
Vink et al. [17]	14.3 ± 4.3	NA	NA	Primary Outcome ARAT Intervention Group: 11.5 ± 16.6 Comparator Group: 13.2 ± 16.8 Secondary Outcome JTHFT Intervention Group: 104.3 ± 28.9 Comparator Group: 97.6 ± 35.8 NHPT Intervention Group: 1.3 ± 3.7 Comparator Group: 2.1 ± 4.9 BI Intervention Group: 12.6 ± 3.9 Comparator Group: 12.3 ± 4.5 JTHFT Intervention Group: 104.3 ± 28.9 Comparator Group: 97.6 ± 35.8 mRS (median, IQR) Intervention Group: 4 ± 1 Comparator Group: 4 ± 1 Stroke Upper Limb Capacity score Intervention Group: 2.9 ± 2.7 Comparator Group: 3.3 ± 3.0	NA	NA	Data were given in graph
Wang et al. [14]	60—90 days	severe	BI	Primary Outcome FMA HF Group: 22.3 ± 8.4 LF Group: 18.0 ± 11.8 Sham Group: 19.2 ± 6.8 Secondary Outcome RMS-SEMG *Exetensor digitorum Median (Quartile) HF Group: 2.26 LF Group: 3.9 Sham Group: 2.10 BI HF Group: 35.0 ± 6.3 LF Group: 31.3 ± 11.6 Sham Group; 31.7 ± 6.71.7			Primary Outcome FMA HF Group: 33.7 ± 9.8 LF Group: 22.7 ± 12.7 Sham Group: 22.0 ± 8.1 Secondary Outcome RMS-SEMG *Exetensor digitorum Median (Quartile) HF Group: 4.31 LF Group: 5.8 Sham Group: 4.11 BI HF Group: 46.7 ± 6.2 LF Group: 35.7 ± 11.9 Sham Group: 33.1 ± 8.4
Wang et al. [24]	2.08 ± 0.71	NA	NA	Only Low CST integrity available FMA-UE High Frequency rTMS: 8.70 ± 1.70 Low Frequency rTMS: 8.56 ± 1.89 Sham rTMS: 8.72 ± 1.77 WMFT High Frequency rTMS: 11.70 ± 1.06 Low Frequency rTMS: 11.33 ± 1.58 Sham rTMS: 11.70 ± 1.73 MBI High Frequency rTMS: 39.00 ± 5.32 Low Frequency rTMS: 40.00 ± 5.33 Sham rTMS: 39.00 ± 5.16	NA	NA	NA (graph)
Wu et al. [8]	14—180 days	NA	NA	FMA-UE rTMS combined with cervical nerve root magnetic stimulation group: 14.80 ± 4.38 rTMS group: 15.47 ± 3.48 cervical nerve root stimulation group: 15.33 ± 3.99 sham group: 14.93 ± 3.94 WMFT rTMS combined with cervical nerve root magnetic stimulation group: 12.53 ± 3.68 rTMS group: 12.27 ± 3.90 cervical nerve root stimulation group: 11.60 ± 4.58 sham group: 12.33 ± 4.67 MBI rTMS combined with cervical nerve root magnetic stimulation group: 36.13 ± 9.47 rTMS group: 35.33 ± 7.16 cervical nerve root stimulation group: 36.67 ± 7.11 sham group: 35.87 ± 6.66	NA	NA	FMA-UE rTMS combined with cervical nerve root magnetic stimulation group: 38.80 ± 3.78 rTMS group: 34.73 ± 5.48 cervical nerve root stimulation group: 33.53 ± 5.95 sham group: 30.70 ± 5.65 WMFT rTMS combined with cervical nerve root magnetic stimulation group: 40.07 ± 6.41 rTMS group: 35.93 ± 4.06 cervical nerve root stimulation group: 33.27 ± 4.09 sham group: 31.87 ± 4.31 MBI rTMS combined with cervical nerve root magnetic stimulation group: 87.07 ± 6.69 rTMS group: 83.67 ± 7.42 cervical nerve root stimulation group: 81.67 ± 6.80 sham group: 78.47 ± 7.76
Yang et al. [10]	30—180 days	NA	NA	JTHFT HF rTMS + hand grip training M1: 399 ± 256 HF rTMS: 323 ± 254 Hand grip training: 373 ± 238 Grip Force Scaling HF rTMS + hand grip training M1: 58 ± 47 HF rTMS: 45 ± 43 Hand grip training: 58 ± 45 FMA-UE HF rTMS + hand grip training M1: 47 ± 6 HF rTMS: 47 ± 8 Hand grip training: 47 ± 7	NA	NA	JTHFT HF rTMS + hand grip training M1: 259 ± 200 HF rTMS: 270 ± 235 Hand grip training: 344 ± 229 Grip Force Scaling HF rTMS + hand grip training M1: 33 ± 37 HF rTMS: 37 ± 40 Hand grip training: 56 ± 47 FMA-UE HF rTMS + hand grip training M1: 57 ± 5 HF rTMS: 53 ± 8 Hand grip training: 51 ± 7

*a-tDCS* anodal transcranial direct current stimulation, *BL-tDCS* bilateral transcranial direct current stimulation, *CIMT* constraint induced movement therapy, *CL* contralesional, *c-tDCS* cathodal transcranial direct current stimulation, *IL* ipsilesional, *M1* primary motor cortex, *N.A* not available, *NIBS* non-invasive brain stimulation, *p/s* pulses/session, *RMT* resting motor threshold, *AMT* active motor threshold, *rPNS* repeated peripheral nerve stimulation, *rTMS* repeated transcranial magnetic stimulation, *s-rTMS* sham repeated transcranial magnetic stimulation, *HF* high frecuency, *LF* low frequency, *s-tDCS* sham transcranial direct current stimulation, *tDCS* transcranial direct current stimulation, *FMA-UE* fugl-meyer assessment for upper extremity, *JTHFT* jjebsen-taylor hand function test, *ARAT* action research arm test, *NHPT* nine hole peg test, *NIHSS* national institutes of health stroke scale, *BBT* box block test, *MAS* modified ashworth Scale, *BI* barthel index, *MBI* modified barthel index, *WMFT* wolf motor function test, *mRS* modified rankin scale, *MRC* medical research council scale, *SIS* Stroke Impact Scale, *MMSE* mini-mental state examination, *HDRS* hamilton depression rating scale, *CNRMS* cervical nerve root magnetic stimulation, *CG* control group, *MSO* maximal stimulator output, Data are given as n and mean ± SD

**Table 3:** Significancy analysis of NIBS

Description	(95% CI)	*P*		$Ta{u}^{2}$, ${I}^{2}$, and Chi (*P*)	No. of comparison
rTMS					
All stroke	2.58 (0.18 to 4.97)		0.04	19.05, 99%, 1367.54 (*P* < 0.00001)	13
Subacute	0.82 (0.46 to 1.19)	0.00001		0.05, 21%, 7.57 (*P* = 0.27)	6
Chronic	3.34 (−3.79 to 10.48)	0.36		52.82, 100%, 963.28 (*P* < 0.00001)	4
Type of stroke					
Ischemic	2.47 (0.21 to 4.74)	0.03		4.64, 96%, 73.36 (*P* < 0.00001)	4
Placement					
IL					
CL	1.04 (0.35 to 1.73)	0.003		0.00, 0%, 0.05 (*P* = 0.82)	2
IL + CL	2.87 (0.10 to 5.64)	0.04		21.55, 99%, 1358.79 (*P* < 0.00001)	11
Type of freq					
HF	3.17 (0.11 to 6.24)	0.04		23.93, 99%, 1322,84	10
LF	0.65 (−0.05 to 1.34)	0.07		0.18, 47%, 3.76 (*P* = 0.15)	3
Combined with an intervention	0.66 (0.06 to 1.27)	0.03		0.34, 60%, 12.56 (*P* = 0.03)	6
Risk of Bias					
Low risk of bias	2.07 (0.14 to 5.27)	0.04		20.08, 99%, 1364.61 (*P* < 0.00001)	12
$$Some concerns risk of bia{s}^{a}$$					1
TBS					
All stroke	1.25 (0.01 to 2.49)	0.05		0.57, 54%, 4.34 (*P* = 0.11)	3
Subacute	1.25 (0.01 to 2.49)	0.05		0.57, 54%, 4.34 (*P* = 0.11)	3
$$Type of strok{e}^{b}$$					
Combined with an intervention	1.04 (0.35 to 1.73)	0.003		0.00, 0, 0.05 (*P* = 0.82)	2
Risk of Bias					
Low risk of bias	1.25 (0.01 to 2.49)	0.05		0.57, 54%, 4.34 (*P* = 0.11)	3
$$Some concerns risk of bia{s}^{a}$$					0
tDCS					
$All strok{e}^{b}$					1

(a) Not enough data to study the effects of non-invasive brain stimulation on the sub category

(b) Not enough data to calculate an effect size

*CI* confidence interval, *rTMS* repeated transcranial magnetic stimulation, *TBS* theta burst stimulation, *IL* ipsilesional, *CL* contralesional, *HF* high frequency, *LF* low frequency

Theta burst stimulation (TBS) did not demonstrate a statistically significant effect in subacute stroke patients, with an effect size (ES) of 1.25 (95% CI: 0.01–2.49; *P* = 0.05). Moderate heterogeneity was observed among the included studies (Tau^2^ = 0.57; I^2^ = 54%; Q = 4.34; *P* = 0.11). In contrast, transcranial direct current stimulation (tDCS) combined with additional rehabilitation interventions showed a significant positive effect on motor recovery (ES = 1.04; 95% CI: 0.35–1.73; *P* = 0.003), with no evidence of heterogeneity across studies (Tau^2^ = 0.00; I^2^ = 0%; Q = 0.05; *P* = 0.82). For repetitive transcranial magnetic stimulation (rTMS), the pooled analysis demonstrated a significant improvement in motor outcomes among stroke patients, with an effect size of 2.58 (95% CI: 0.18–4.97; *P* = 0.04). However, substantial heterogeneity was detected (Tau^2^ = 19.05; I^2^ = 99%; Q = 1367.54; *P* < 0.00001). The substantial heterogeneity observed in the rTMS subgroup may be attributable to variations in stimulation parameters, stroke chronicity, and rehabilitation protocols across studies.

### Forest plot data

Visual inspection of the forest plots indicated that most studies favored the intervention group compared with the control group, as reflected by effect estimates located to the right of the line of no effect. However, the wide confidence intervals observed in several studies suggest variability in treatment effects across trials. Figure 3 summarizes a forest plot for the effectiveness of NIBS of fine motor in subacute and chronic stroke patients and Fig. 4 summarizes a forest plot for the effectiveness of assessment of fine motor in FMA and ARAT.

Publication bias was assessed through visual inspection of funnel plots. The funnel plots appeared relatively symmetrical, suggesting no substantial evidence of publication bias among the included studies. However, the limited number of studies in some subgroups may reduce the reliability of this assessment. Overall, the meta-analysis findings suggest that non-invasive brain stimulation—particularly rTMS and combined tDCS interventions—may improve motor recovery in stroke patients, although considerable heterogeneity across studies warrants cautious interpretation of these findings.

## Discussion

This systematic review and meta-analysis evaluated the therapeutic effects of non-invasive brain stimulation on fine motor recovery following stroke. The findings indicate that NIBS, particularly rTMS and tDCS, can significantly enhance motor recovery when used as an adjunct to conventional rehabilitation. In this study, TMS intervention within the NIBS is commonly used due to its effectiveness in treating depression and other conditions that have proven resistant to traditional therapies. Its effectiveness, non-invasive approach, safety record, adaptability, multiple FDA approvals for different conditions, and continual exploration of its potential applications all contribute to its widespread utilization in clinical settings for addressing various neurological and mental health conditions [27]. It shows potential for enhancing motor function impairment in disorders such as Parkinson's disease, multiple sclerosis, and stroke recovery [28].

The observed improvements are likely mediated through mechanisms of activity-dependent neuroplasticity. After stroke, the brain undergoes dynamic reorganization processes aimed at compensating for damaged neural networks. Neuromodulatory interventions such as NIBS may facilitate this reorganization by modulating cortical excitability and strengthening synaptic connectivity within motor networks [29]. NIBS has been shown to induce it, which plays a crucial role in motor learning (ML) capacity, some studies have found that cortical excitability changes induced in M1 by tDCS and iTBS may be related to reaction time and retention of newly acquired skills, but the physiological significance of these results is uncertain [28]. One key mechanism underlying NIBS-induced recovery involves the restoration of interhemispheric balance. Stroke often disrupts the equilibrium between the affected and unaffected hemispheres, resulting in excessive inhibitory signals from the contralesional hemisphere. By either enhancing excitability in the ipsilesional motor cortex or suppressing contralesional inhibition, NIBS may promote functional reactivation of motor circuits. Other studies also supported this statement representing that NIBS appears to positively affect motor function by enhancing cortical excitability of stroke patients [6, 30, 31].

Our findings suggest that rTMS demonstrated the most consistent therapeutic effects among the evaluated interventions. This observation aligns with previous neurophysiological studies showing that rTMS can induce long-lasting changes in cortical excitability and synaptic plasticity. High-frequency stimulation applied to the ipsilesional hemisphere may enhance excitatory activity, whereas low-frequency stimulation over the contralesional hemisphere may reduce maladaptive inhibition [3–8, 12, 24], Quo, 2020, [10, 18, 24, 25], which is later associated with improvement of neural plasticity [7–10]. Improvement of neural plasticity will result in fine and gross motor refinement, which is in line with our finding with 12 of them enhancing upper limb motor recovery[3, 5, 6, 7, 8, 12, 13, 18];Quo, 2020, [17, 21, 25] and 2 of them advancing hand impairment [16, 23], also improve attention function post-stroke in one of the studies [23]. Fascinatingly, one study showed decreasing in motor function compared to sham group [20]. This might be the result of combining NIBS intervention with fluoxetine because this group exhibited a rise in intracortical facilitation in the unaffected hemisphere, a change that corresponded with the diminished motor advantages [27]. Among the interventions explored, TMS stands out as a potential method for enhancing fine motor abilities in stroke patients. Several research investigations have documented the neurophysiological alterations observed in individuals who have experienced strokes following the application of NIBS utilizing TMS methods. However, the frequency and duration of TMS sessions conducted in each trial may vary, potentially influencing the outcomes for patients. rTMS and tDCS therapies operate according to the interhemispheric competition theory, and they can alter brain excitability, foster neuroplasticity, and enhance motor skills [32]. Interestingly, the present analysis suggests that chronic stroke patients may still benefit from neuromodulation, challenging the traditional assumption that meaningful recovery is limited to the early post-stroke period. This finding highlights the potential of neuromodulatory therapies to reactivate latent neural networks even in later stages of recovery [13, 15, 18, 33, 34, 43], except in several studies using cathodal tDCS and bilateral tDCS as an intervention [35, 47]. Therefore, this brand-new finding can be a new approach to therapeutic interventions for chronic patients.

The discrepancies in findings may be explained by several reasons. Initially, there is ongoing debate regarding the existence of a genuine "imbalance" in interhemispheric inhibition in the human brain following a stroke, as indicated by recent evidence [36]. Additionally, the interhemispheric inhibition model is considered overly simplistic, and the impact of rTMS (repetitive transcranial magnetic stimulation) may differ based on various factors [37]. Further investigation is necessary to draw a definitive conclusion regarding the comparative efficacy of inhibitory rTMS in influencing cortical excitability and promoting motor recovery in patients with subacute versus chronic strokes [38].

Comparison between stroke subtypes could not be performed because none of the included studies exclusively investigated patients with hemorrhagic stroke. Most studies primarily involved patients with ischemic stroke, which limited the possibility of conducting subgroup analyses based on stroke type. Nevertheless, the pooled analysis suggested that repetitive transcranial magnetic stimulation (rTMS) produced significant improvements in motor recovery among patients with ischemic stroke, with an effect size (ES) of 2.47 [42, 43]. Furthermore, several studies reported that high-frequency rTMS produced greater therapeutic effects compared with low-frequency stimulation. High-frequency stimulation is generally believed to enhance cortical excitability in the ipsilesional motor cortex, thereby facilitating motor recovery after stroke [44, 45]. These findings are consistent with previous studies, including those reported by Ibrahim [4], Wang [24], Wu [8], Yang [10], Ke [21], Liu [23], and Wang [24], which demonstrated significant improvements in motor function following high-frequency rTMS interventions. Similar conclusions were also reported in earlier research by Caglayan [39].

Fugl-Meyer Assessment (FMA) is frequently preferred over the ARAT because of its robust psychometric support, reliability, and validity when evaluating upper limb functionality across different groups of patients, including those who have had strokes to utilize in clinical and research [24]. Combined rehabilitation approaches were predominantly applied to middle-aged and older adults, reflecting the higher prevalence of stroke in these age groups. Regarding outcome measures, studies evaluating resistance and functional training commonly used standardized clinical assessments such as the Fugl-Meyer Assessment (FMA), the Wolf Motor Function Test (WMFT), and range-of-motion measurements to evaluate improvements in upper-limb motor function. For aerobic-based rehabilitation interventions, the most frequently reported outcome measures included gait parameters and the Six-Minute Walk Test (6MWT), which assess walking capacity and functional mobility. Importantly, the clinical stage and time elapsed since stroke onset do not necessarily reflect the severity of motor impairment. Therefore, this study aimed to analyze rehabilitation interventions used in stroke survivors and to identify appropriate exercise approaches according to the patient’s stage of recovery [40]. In several studies, the active intervention group demonstrated significantly greater improvements than the sham group across multiple outcome measures. Significant differences were observed in the Fugl-Meyer Assessment (FMA), including motor function and joint pain components, as well as in the Wolf Motor Function Test (WMFT), particularly in functional ability and the weight-to-box task (*p* < 0.05). The improvements also exceeded the minimal clinically important difference (MCID). The between-group difference was 4.9 points for the FMA (95% CI: 0.007–9.799) and 6.54 points for the WMFT (95% CI: 1.10–14.15). Regarding secondary outcomes, significant ents were also observed in activities of daily living (ADL) independence, measured using the Functional Independence Measure (FIM), with a between-group difference of 8.63 points (95% CI: 1.37–18.64). Additionally, patients in the active intervention group reported greater perceived recovery in quality of life at the 90-day follow-up (*p* < 0.05) [22].

Following a stroke, patients typically exhibit reduced cortical excitability in the ipsilesional primary motor cortex (M1), accompanied by increased transcallosal inhibition from the contralesional to the ipsilesional hemisphere compared with healthy individuals [46, 47]. This imbalance in interhemispheric activity is reflected by reduced motor evoked potentials (MEPs) and a prolonged ipsilateral silent period (iSP). Application of dual transcranial direct current stimulation (tDCS) has been shown to modulate this imbalance by increasing MEP amplitudes and shortening iSP duration in the ipsilesional M1 of stroke survivors. These changes suggest a restoration of cortical excitability and interhemispheric balance. Furthermore, the magnitude of MEP enhancement following tDCS appears to correlate with the baseline ratio of transcallosal inhibition between the contralesional and ipsilesional hemispheres, as measured by the iSP. This finding suggests that the baseline level of interhemispheric imbalance may influence individual responsiveness to neuromodulatory interventions [26, 40].

Fine motor function can be impaired following subacute stroke, particularly when the basal ganglia are involved. Lesions affecting the basal ganglia play an important role in disrupting motor control circuits responsible for coordination and precision of voluntary movements. Previous studies have shown that ischemic stroke involving the basal ganglia, including cases reported in pediatric populations, can lead to significant impairments in fine motor performance and hand dexterity. [Li, 2021; [41].

Another important observation is that combined interventions yielded greater therapeutic effects than NIBS alone. Multimodal rehabilitation approaches that integrate neuromodulation with task-specific motor training may provide synergistic benefits by coupling cortical stimulation with functional motor learning. TMS proves to be a reliable and secure method for exploring this area of study, holding considerable promise for rehabilitation purposes [36]. Overall, the meta-analysis findings suggest that non-invasive brain stimulation—particularly rTMS and combined tDCS interventions—may improve motor recovery in stroke patients, although considerable heterogeneity across studies warrants cautious interpretation of these findings. However, substantial heterogeneity across studies remains a major limitation in the current evidence base. Differences in stimulation parameters, treatment duration, patient characteristics, and outcome measures complicate direct comparisons between studies. Standardization of stimulation protocols will be essential for future clinical translation.

### Limitation of the study

This study have several limitations should be considered. First, heterogeneity among the included studies was relatively high, particularly regarding stimulation protocols and outcome measures. Second, many studies involved relatively small sample sizes, which may limit the statistical power of pooled analyses. Third, long-term follow-up data were limited, making it difficult to determine the sustainability of treatment effects.

## Conclusion

This systematic review and meta-analysis demonstrates that non-invasive brain stimulation is a promising adjunctive therapy for improving fine motor recovery after stroke. Both rTMS and tDCS appear to facilitate motor recovery by modulating cortical excitability and promoting neuroplastic reorganization. Among the evaluated techniques, rTMS showed the most consistent therapeutic effects, particularly when combined with conventional rehabilitation strategies. Despite encouraging findings, further large-scale randomized controlled trials are required to establish optimal stimulation parameters, identify patient populations most likely to benefit, and evaluate long-term functional outcomes. Advancing our understanding of neuromodulation-based rehabilitation may contribute to the development of more effective and personalized treatment strategies for stroke survivors.

## Data Availability

All data generated or analysed during this study are included in this published article.
